# Evaluation de la sécurité du circuit des médicaments anticancéreux dans un hôpital régional en Tunisie

**DOI:** 10.11604/pamj.2016.23.30.8600

**Published:** 2016-02-04

**Authors:** Jihène Sahli, Meriam El Ghardallou, Iheb Bougmiza, Besma Henchiri, Manel Limam, Rim Mejdoub, Ali Mtiraoui, Thouraya Ajmi

**Affiliations:** 1 Laboratoire de Recherche « LR12ES03 », Département de Médecine Familiale et Communautaire, Faculté de Médecine Ibn El Jazzar Sousse, Université de Sousse,Tunisie; 2 Service de Carcinologie, Hôpital Régional de Gafsa, Tunisie

**Keywords:** Risk management, anticancer drugs, patient safety, Risk management, anticancer drugs, patient safety

## Abstract

**Introduction:**

Parmi les événements indésirables, ceux liés au circuit des médicaments occupent une place importante et risquent de causer un préjudice grave aux patients. Dans ce contexte, nous avons mené cette étude dans l'optique de décrire et d’évaluer le circuit des médicaments anticancéreux dans un hôpital régional tunisien.

**Méthodes:**

Il s'agit d'une étude évaluative du risque lié aux médicaments anticancéreux, type « visite de risque » menée sur une période de 15 jours au cours de l'année 2014 au service de cancérologie de l'hôpital régional de Gafsa (Tunisie). Cette méthode d’évaluation est inspirée de celle conduite par le projet « SECURIMED » développé par le Comité de Coordination de l'Evaluation Clinique et de la Qualité en Aquitaine (CCECQA), en France.

**Résultats:**

Dans notre étude, l'observation du circuit des médicaments anticancéreux a révélé certaines insuffisances. On a noté que la répartition des missions des différents acteurs est sujette parfois à des glissements de tâches. Un manque important ainsi que une inadéquation aux normes au niveau des équipements nécessaires pour la préparation et la protection des professionnels manipulant ces médicaments ont été également décelés.

**Conclusion:**

La sécurisation du circuit des médicaments devrait être une priorité inscrite dans l'ensemble des démarches nationales et partagée par tous les intervenants et ce dans l'optique d'atteindre un objectif prémium: la qualité de la prise en charge globale et la sécurité des patients.

## Abstract (en)

**Introduction:**

Nowadays, the circuit of drugs is a plague. This situation may cause serious harm to patients. In this context, we conducted a study with the aim to describe and evaluate the circuit of anticancer drugs in a Tunisian regional hospital.

**Methods:**

This is an evaluative study of the risk of anticancer drugs, conducted over a period of 15 days during the year 2014 in the Department of Cancer Research of the Regional Hospital of Gafsa (Tunisia). The evaluation method is based on that conducted by the project "SECURIMED" and developed by the Coordination Committee of the Clinical Evaluation and Quality in Aquitaine (CCECQA) in France.

**Results:**

In our study, the observation of anticancer drugs circuit has revealed some deficiencies. We noted that the roles of the various actors are subject sometimes to tasks shifting, which may sometimes be dangerous. The study also revealed a lack and an inadequacy with the standards in terms of the necessary equipment for the preparation of the anticancer drugs.

**Conclusion:**

Securing drugs circuit should be a priority included in all national processes and shared by all stakeholders to achieve a premium goal: the quality of care and patient safety.

## Introduction

La majorité des systèmes de santé dans le monde font face actuellement au défi majeur de la qualité et la sécurité des soins [1]. En faisant référence à l'Institute Of Medicine (IOM), la sécurité des soins fait partie des cinq dimensions essentielles de la qualité des soins (l'efficacité, la sécurité, la réactivité, l'efficience et l'accessibilité). Cette dimension est basée sur le principe de ne pas nuire aux patients [2]. La littérature montre que l'incidence des événements indésirables (EI) liés aux soins est en moyenne de l'ordre de 10% avec une haute évitabilité d'au moins 50% [1]. Parmi ces événements indésirables, ceux liés au circuit des médicaments occupent une place importante et risquent de causer un préjudice grave aux patients [3]. Ce préjudice varie de l'allongement de la durée de séjour à l'hôpital jusqu’à l'augmentation de la morbi-mortalité [4]. Le circuit des médicaments dans un établissement de santé recouvre la prescription, l'analyse et la validation de cette prescription, la préparation, la livraison, la distribution, le transport, la détention et l'administration du médicament, l'information du patient, la surveillance du traitement, les commandes par la pharmacie, l'analyse de l'activité, la gestion des périmés et des retraits de lots [5]. Il s'agit d'un processus complexe combinant des étapes pluridisciplinaires et interdépendantes visant un objectif commun: l'utilisation sécurisée, appropriée et efficiente du médicament chez le patient pris en charge en établissement de santé [5]. Cette complexité est source d'erreurs [6]. Parmi les médicaments en milieu hospitalier, et devant l'augmentation considérable de l'incidence des cancers, les anticancéreux sont devenus d'un usage de plus en plus fréquent. Cette entité de médicaments suscite un intérêt particulier du fait du risque qu'elle engendre aussi bien pour les patients que pour le personnel soignant au cours du processus de son utilisation [7]. En Tunisie, rares sont les études évaluant la sécurité du circuit des médicaments d'une façon générale et ceux des anticancéreux en particulier. Dans ce contexte, nous avons mené cette étude dans l'optique de décrire et d’évaluer le circuit des médicaments anticancéreux au service de cancérologie de l'Hôpital Régional de Gafsa (HRG).

## Méthodes

### Type et lieu de l’étude

Il s'agit d'une étude évaluative du risque lié aux médicaments anticancéreux, type « visite de risque » menée sur une période de 15 jours au cours de l'année 2014 au service de cancérologie de l'hôpital régional de Gafsa. Il s'agit d'un hôpital public régional (2 ^ème^ niveau) comportant 20 services et faisant l'objet de près de 16000 admissions par an. Le service de cancérologie est un service fonctionnel depuis 3 ans. Le choix de ce service comme lieu d’étude était fait de manière raisonnée du fait de l'utilisation de médicaments à haut risque (anticancéreux). La visite de risque (ou de site) combine plusieurs outils d’évaluation: consultation de documents, questionnaires standardisés, entretiens avec les acteurs du circuit du médicament, une observation en fonctionnement des systèmes étudiés et la réalisation des différentes mesures sur site. Cette méthode d’évaluation est inspirée de celle conduite auparavant par le projet « SECURIMED ». Ce dernier a été développé par le Comité de Coordination de l'Evaluation Clinique et de la Qualité en Aquitaine (CCECQA), en France [8]. Dans notre travail cinq étapes étaient menées successivement. **Première étape:** présentation du groupe de travail de la visite de risque. **Deuxième étape:** consultation des documents disponibles au niveau du service et portant sur la sécurité du circuit du médicament. **Troisième étape:** observation du circuit des anticancéreux par le groupe de la visite. Cette étape avait évalué les vulnérabilités et les défenses à l'aide de deux types de check-list: une pour les services cliniques et la seconde pour le service de pharmacie. **Quatrième étape:** entretiens individuels avec les professionnels. Ces entretiens ont été effectués auprès des médecins, des pharmaciens et d'infirmiers présents lors de la visite à l'aide de questionnaires spécifiques pour chaque catégorie professionnelle afin de connaitre les vulnérabilités et les défenses perçues par les professionnels et leurs attitudes. **Cinquième étape:** restitution à la fin de la visite de risque. Au cours de cette étape, les principales défenses identifiées, les défenses absentes ou présentes mais non opérationnelles, et les perspectives d'amélioration ont été communiquées aux professionnels du service ***Groupe de travail***. Cette visite de risque a été menée par un groupe de travail pluridisciplinaire formé par deux médecins spécialistes en cancérologie, deux pharmaciens et une technicienne supérieure en sciences infirmières. ***Analyse statistique***. Une analyse descriptive des résultats des entretiens avec les professionnels de la santé et des observations du circuit du médicament a été effectuée.

## Résultats

### Description de l'organisation générale du service de cancérologie

Le service de cancérologie de l'hôpital régional de Gafsa est classé niveau III d'exposition. Deux types de classement des niveaux d'exposition précisés dans les recommandations du Centre National d'Information sur les Médicaments Hospitalier (CNIMH) (en France) [9] sont admis. En pratique, il existe un regroupement de ces deux classifications, aboutissant chacune à trois niveaux d'exposition à prendre en termes de locaux, d’équipement, de matériels et de protections individuelles. Le niveau I correspond à la préparation et l'administration occasionnelle, le niveau II correspond à la préparation et l'administration en quantité modérée et le niveau III correspond à la préparation et l'administration de façon intensive [10].

#### Le personnel

le staff du service de cancérologie de l'HRG était formé de deux médecins spécialistes en carcinologie et exerçant depuis deux ans et demi dans cette structure ainsi que dix infirmiers (9 femmes + 1 homme). Cette équipe collaborait avec deux pharmaciens, exerçant depuis cinq ans dans cet hôpital. Ces derniers étaient affectés à la pharmacie de l'hôpital. Tous les infirmiers travaillant au sein du service n'ont jamais eu de formation spécialisée en matière de cancérologie, ni avant d’être affectés au service ni au cours de leur fonction actuelle. Quarante pour cent du personnel n’était pas qualifié pour son poste. Les aides-soignants faisaient fonction d'infirmiers et ceci par manque de personnel. Un seul infirmier parmi le personnel du service de cancérologie de Gafsa (SCG) avait une tenue conforme aux protocoles de bonne pratique.

#### Les locaux

On a noté la présence d'une salle propre conçue pour la préparation des médicaments anticancéreux.

#### Les équipements

Le service de cancérologie ne disposait pas de hotte à flux laminaire et seul un isolateur était mis à la disponibilité du personnel de ce service. Dans toutes les chambres, on a noté l'absence de lavabos qui étaient présents uniquement dans les vestiaires. Un réfrigérateur et un système de rangement (armoires) étaient présents au niveau de la salle de préparation des médicaments. Parmi les accessoires de protection, seuls les gants propres et les bavettes étaient disponibles. On a également constaté un manque au niveau de certains équipements tels que les lunettes de protection, les gants stériles et les sur-blouses.

#### Résultats de la consultation des documents

Les principaux supports de prescription des médicaments étaient des ordonnances médicales, et des supports de prescription pour les infirmiers. Par ailleurs, on n'a pas constaté l'existence de pancartes de signalisation de la dangerosité des produits manipulés au niveau du service ni d'affiches indiquant les protocoles de la préparation des médicaments.

### Résultats de l'audit clinique du circuit des anticancéreux

**Figure 1:**
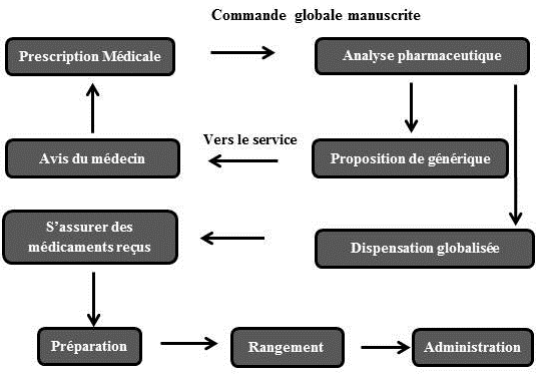
Représentation du circuit des anticancéreux à l'hôpital régional de Gafsa

La Figure 1 récapitule le circuit des anticancéreux au service de cancérologie de l'HRG. Au total, onze observations ont été effectuées, réparties entre le service clinique et la pharmacie de l'hôpital. Lors de l'observation des conditions matérielles de prescription, on a noté certaines insuffisances notamment: absence totale de documentation d'information (liste ou livret mis à jour des médicaments dans l’établissement, dictionnaire Vidal, protocoles thérapeutiques) pour les médecins prescripteurs, absence de pharmacien ou de préparateur en pharmacie au sein du service, absence de relecture systématique de l'ordonnance particulièrement après interruption du médecin lors de sa prescription, absence de séparation des médicaments look alike, sound alike (LASA). Les résultats de l'observation des différentes phases du circuit du médicament anti-cancéreux sont résumés dans le Tableau 1 et le Tableau 2.

**Tableau 1:** Résultat de l'observation des différentes phases du circuit des médicaments anti-cancéreux (phases de prescription, transmission, et dispensation)

Phase observée	Oui/non
**Phase de prescription**	
Identification du patient	
Nom et prénom	Oui
Matricule	Oui
Numéro du lit	Oui
Age du patient	Non
Poids du patient	Oui
Identification du médecin prescripteur	Oui
Identification du service	Oui
Identification du médicament	Oui
Informations sur le dossier du patient	Non
Livret des médicaments disponibles (mis à jour)	Non
Protocoles thérapeutiques (mis à jour)	Non
Abréviations dangereuses	Non
**Phase de transmission à la pharmacie**	
Transmission nominative	Oui
Transmission à la pharmacie des informations sur le patient (diagnostic, allergies)	Non
Abréviations dangereuses	Non
**Phase de dispensation / délivrance**	
Listes mise à jour des prescripteurs autorisés	Oui
Vérification de la cohérence de la prescription (redondance, incompatibilité physico-chimique…)	Oui
Liste mise à jour des médicaments disponibles dans la pharmacie (et/ou équivalents)	Oui
Analyse pharmaceutique règlementaire (manuellement)	Oui
Dispensation journalière individualisée	Oui
Liste mises à jour des dotations des services	Oui
Préparation des médicaments anticancéreux injectables	Non 9 observations /11(81,8%)
Conditions matérielles à la pharmacie	
Accessibilité/ Signalisation	Oui
Conditions de stockage	
Contrôle de la température	Oui
Stockage séparé des médicaments de consonance semblable ou d'apparence semblable « médicaments look-alike, sound-alike (LASA)	Non

**Tableau 2:** Résultat de l'observation des différentes phases du circuit des médicaments anti-cancéreux (phases de transport vers le service, réception et stockage, préparation et administration)

**Transport vers le service de carcinologie**	
Respect de la chaine de froid	Oui
Rangement adéquat	Non
**Réception/Stockage au SCG**	
Notification de l'horaire de réception	Non
Surfaces adaptées	Oui
stockage séparé des médicaments de consonance semblable ou d'apparence semblable « médicaments look-alike, sound-alike (LASA)	Non
**Préparation des médicaments**	
Préparation exclusive par les infirmiers	Non
Préparation dans le local de préparation	Non (80%)
Double contrôle de la préparation	Non
Remise des médicaments non utilisés à la pharmacie	Non (55,5%)
**Administration du médicament**	
Vérification de l'identité du patient	Oui
Vérification de la date de préemption, de l'intégrité de l'emballage et l'apparence du médicament	Oui
Contrôle des allergies	Non
Administration du médicament par son préparateur	Non
Equipements toujours disponibles pour l'administration (matériel de perfusion)	Non
Enregistrement de l'administration du médicament (document nominatif)	Oui
Transmission de l'information au patient concernant son traitement	Non (5 cas/11)
Remise de documents clairs au patient sur le traitement	Non (6 cas sur 11)
Alerte du médecin des effets indésirables liés aux médicaments au moment de détection de cet effet (Le signalement aux médecins hors service se faisait par les infirmiers par téléphone)	Oui

### Résultats des entretiens avec les professionnels de la santé

Au total, dix entretiens avec les infirmiers travaillant dans le SCG ont été effectués. Le nombre de patients bénéficiant des ordonnances de médicaments était estimé entre 15 à 20 patients par jour. Le nombre de médicaments prescrits par patient était estimé entre 3 à 4. L'ordonnance nominative manuscrite du médecin ou une recopie manuscrite par l'infirmier sont les seuls supports reportés par les infirmiers comme moyens à partir des quels ils vont préparer et administrer les médicaments anticancéreux. Le recopiage se fait sur la feuille de température et sur le cahier de l'infirmier. La moitié des infirmiers interrogés (50%) juge comme non sécurisé, ce support de prescription. Les deux médecins spécialistes travaillant dans le SCG ont participé aux entretiens. Parmi les renseignements reconnus par les médecins comme non signalés lors de la prescription, on cite l’âge du patient. Deux entretiens ont été fait avec le personnel de la pharmacie (pharmacien et préparateur). Les deux sujets interrogés ont reconnu que l’âge du patient est la seule information reconnue comme manquante à l'ordonnance nominative, lors de la délivrance du médicament. Ils ont déclaré que les ordonnances transmises sont toutes analysées à la pharmacie par l'agent présent (pharmacien ou préparateur) et ceci le jour même de sa réception. Les interactions médicamenteuses étaient déclarées comme recherchées systématiquement. Le Tableau 3 récapitule le résultat de l'entretien avec les professionnels de la santé.

**Tableau 3:** Résultats des entretiens avec les professionnels de santé

Réponses des professionnels de la santé	Oui	Non
**Réponses des médecins (2)**		
Reconnaitre la prescription des anticancéreux comme un acte important	2	0
Reconnaitre le support de prescription (ordonnance papier) comme non sécurisé	1	1
Faire des prescriptions orales ou téléphoniques	1	1
Vérification de la disponibilité du médicament dans l’établissement	2	0
Vérification de la bonne compréhension de la prescription par les infirmiers	2	0
Reconnaitre la survenue d'erreurs médicales dans le SCG	0	2
Impossibilité (ou probabilité très faible) de survenue d'erreurs médicales	2	0
**Réponses des infirmiers (10)**		
Reconnaitre le support de prescription [ordonnance nominative du médecin / copie manuscrite par les infirmiers] comme insuffisant	5	5
Reconnaitre la préparation et l'administration des médicaments anticancéreux comme des actes importants de l'activité	10	0
Administration sans prescription	0	10
Possibilité d'administration par prescription téléphonique	2	8
Informer le médecin sur le médicament de remplacement proposé par le pharmacien	10	0
Vérification de la date de préemption des médicaments	10	0
Recours à une source d'information sur les médicaments anticancéreux	3	7
**Réponses des pharmaciens (2)**		
Connaitre les supports de prescription de l'HRG	2	0
Reconnaitre le support de prescription des anticancéreux comme non sécurisé	2	0
Les éléments qui figurent dans la prescription des anti-cancéreux sont:		
Nom du patient	2	0
Age	0	0
Sexe	2	0
Voie d'administration	2	0
Durée du traitement	2	0
Nom et signature du prescripteur	2	0
Analyse systématique des prescriptions (pharmacien / préparateur)	2	0
Recherche systématique d'interactions médicamenteuses	2	0
Reconnaitre l'absence de pharmaciens dans certaines périodes (Jours fériés, horaires de fermeture de la pharmacie) comme élément défaillant	2	0
Reconnaitre le transport des anticancéreux au SCG comme sécurisé	2	0
Reconnaitre la chaine de froid comme sécurisée	2	0
Reconnaitre les sources d'information sur les médicaments anti-cancéreux comme disponibles et sécurisés	2	0

## Discussion

Notre étude est parmi les rares études menées en Tunisie ayant pour but d'explorer les conséquences de la prescription des médicaments en termes de sécurité pour les patients. Dans notre travail, le suivi du circuit des médicaments a été motivé par le résultat des études antérieures qui ont démontré la fréquence élevée des événements indésirables liés aux médicaments et leurs graves conséquences pour les patients et en termes de coûts de santé [3]. Elle nous a permis de faire un état des lieux sur la sécurité du circuit des médicaments anticancéreux dans un service de cancérologie intégré dans un hôpital de deuxième niveau. Ce travail a montré que le système actuel du circuit des médicaments ne permet pas de minimiser les risques pour la sécurité des patients et que ce système dans sa globalité nécessite des améliorations. L'identification de ces défaillances s'est basée sur une méthode scientifiquement prouvée en matière d’évaluation des pratiques, qui est la visite du risque. Cette méthode permet d'appréhender les facteurs humains, de comprendre la chaîne des événements et d'analyser les causes des problèmes identifiés [11]. Cette approche a le mérite d'aller à la rencontre des professionnels, d’être à leur écoute, de les impliquer dans la démarche de gestion des risques, d'introduire une culture de risques, de faciliter la communication entre les différents acteurs d'un même système [8]. Cependant, le service choisi a été informé à l'avance à propos de la visite de risque, ce qui peut induire un changement des comportements du personnel observé. Dans notre étude, on a noté que la répartition des missions des différents acteurs est sujette parfois à des glissements de tâches qui risquent d’être parfois dangereux. En effet, on a constaté que par moment, des aides-soignants effectuent des tâches d'infirmiers. Ainsi, en plus du manque de formation spécialisée que doit recevoir les professionnels de ce service, s'ajoute l’éloignement de chaque acteur à sa mission prioritaire. En référence au guide de la Haute Autorité de Santé « Outils de sécurisation et d'auto-évaluation et de l'administration des médicaments» le glissement des tâches figurent parmi les causes des erreurs médicamenteuses [12].

Un manque important ainsi qu'une inadéquation aux normes au niveau des équipements nécessaires pour la préparation et la protection des professionnels manipulant ces médicaments ont été également décelés. Ce même constat a été rapporté dans d'autres études tunisiennes comme celle de Triki et al, menée au service d'oncologie médicale du centre hospitalo-universitaire Habib Bouguiba de Sfax et qui a conclu à ce que seulement 70% des infirmières portaient des gants en latex lors de la préparation des cytotoxiques et 40% portaient un masque. Par ailleurs, l'observation du circuit de l'Endoxan a dévoilé de nombreuses défaillances [13]. La prescription manuscrite des traitements et par la suite leurs transcriptions au niveau des fiches de prescription des infirmiers exposent à un risque d'erreurs important. Ces erreurs sont néanmoins souvent évitables [14, 15]. Au cours de la transcription, la dose, l'unité, la voie d′administration, et la durée d′administration peuvent être modifiées [15]. Dans notre étude, bien qu'il y ait une vérification et une analyse pharmaceutique des médicaments demandés, on a noté un manque de communication entre le pharmacien et les médecins prescripteurs avant la prescription du traitement, un manque d'informations portant sur les renseignements cliniques du patient sur la prescription adressée au pharmacien et la non disponibilité du pharmacien pendant des plages horaires journalières et même pendant des journées entières comme les jours fériés. Ceci met à risque la sécurité des patients. Un tel constat a été retrouvé dans l’étude de El Mhamdi et al [16].

Dans notre étude, on a remarqué l'absence de séparation des médicaments dont le nom ou l'apparence prêtent à confusion ou les médicaments look alike, sound alike (LASA). Certaines données révèlent que les confusions entre différents noms de médicaments représentent environ un quart des erreurs en lien avec leur utilisation [17]. Certaines interventions impliquant une approche multidisciplinaire dans l'acquisition des médicaments associant médecins, pharmaciens, professionnels de la santé, dirigeants d'hôpitaux, agences d'accréditation des médicaments et industries ont fait la preuve de leur efficacité pour mieux sécuriser l'utilisation des médicaments et devraient être promulguées par tous les intervenants, depuis la fabrication des médicaments jusqu’à leur administration aux patients [18]. Le circuit du médicament est un processus complexe impliquant plusieurs acteurs et chaque acteur doit contribuer dans une logique d'enchaînement à sécuriser le processus [8]. La collaboration des pharmaciens avec l′équipe médicale contribue à une réduction significative des évènements indésirables liés aux médicaments jouant ainsi un rôle crucial dans la promotion de la sécurité des médicaments. C'est ainsi que dans l’étude de Kucukarslan et al [19], la présence d'un pharmacien dans l'unité médicale réduit de 78% les événements indésirables liés aux médicaments. L'IOM affirme que les pharmaciens devraient être inclus pendant le processus de prescription et ce dans le cadre d'une stratégie visant à améliorer la sécurité des médicaments [2]. D'un autre côté, il convient de compléter et renforcer la formation initiale des professionnels de santé en termes de connaissances et compétences à acquérir en vue d'améliorer la sécurité des patients [20]. La gestion du circuit du médicament nécessite une approche participative se basant sur une collaboration entre les différents acteurs. Une approche privilégiant la communication peut être garante d'une administration médicamenteuse sûre et en temps opportun [21]. C'est ainsi que les résultats de ce travail doivent être présentés aux différents acteurs impliqués dans la gestion du circuit des médicaments anticancéreux afin de les engager à la recherche de solutions adéquates et adaptées au contexte non dénué de contraintes.

## Conclusion

La sécurisation du circuit des médicaments devrait être une priorité inscrite dans l'ensemble des démarches nationales et partagée par tous les intervenants et ce dans l'optique d'atteindre un objectif prémium: la qualité de la prise en charge globale et la sécurité des patients. De ce fait, des dispositions réglementaires et un cadre juridique doivent être mis en place pour garantir l'atteinte de cet objectif prioritaire.

### Etat des connaissance sur le sujet

- La revue de la littérature montre que l'incidence des événements indésirables (EI) liés aux soins est en moyenne de l'ordre de 10% avec une haute évitabilité d'au moins 50% et que en moyenne 44 000 à 98 000 décès sont attribuables chaque année aux erreurs médicales. Parmi ces erreurs, celles liées aux cytotoxiques sont fréquentes et particulièrement dangereuses en raison du potentiel hautement toxique des médicaments concernés, d'où l'intérêt croissant de la sécurisation de ce circuit.

### Contribution de notre étude a la connaissance

- Notre étude ayant trait à la sécurité des patients et à la qualité des soins, est parmi les rares études en Tunisie qui ont porté sur l’évaluation du circuit des médicaments et en particulier celui des anticancéreux. De plus et à notre connaissance, notre étude est la première à avoir utilisé la méthode de la visite du risque pour évaluer le circuit des anticancéreux en Tunisie.
